# Gut microbiota in coronary artery disease: a friend or foe?

**DOI:** 10.1042/BSR20200454

**Published:** 2020-05-15

**Authors:** Bo Zhang, Xinxin Wang, Ran Xia, Chunsheng Li

**Affiliations:** 1 Department of Geriatrics, The Affiliated Hospital to Changchun University of Chinese Medicine, Changchun, China; 2 Medical Record Room, The Affiliated Hospital to Changchun University of Chinese Medicine, Changchun, China; 3 Department of Gastrointestinal Colorectal and Anal surgery, China-Japan Union Hospital of Jilin University, Changchun, China

**Keywords:** coronary artery disease, gut microbiota, heart, ischemia, trimethylamine-N-oxide

## Abstract

There is a growing interest in the role of gut microbiota in the pathophysiology of several diseases, including coronary artery diseases (CAD). Gut microorganisms may produce beneficial effects in myocardial ischemia either directly in the form of exogenous administration or indirectly by acting on fiber-rich food to produce important cardioprotective components. The harmful effects of gut microbiota in CAD are due to alteration in their composition with a significant decrease in *Bacteroidetes* and an increase in *Firmicutes, Escherichia, Shigella*, and *Enterococcus*. The altered microbiota may produce potentially toxic metabolites, including trimethylamine-N-oxide (TMAO). Indeed, the fasting plasma levels of TMAO are directly correlated to increased risk of major cardiovascular events in CAD patients, and it is proposed as a potential biomarker to predict the onset of major cardiovascular events. It is concluded that the change in the composition of gut microbiota in CAD patients may predispose to more harmful effects. However, exogenous delivery of probiotics may overcome the detrimental effects of myocardial ischemia.

## Introduction

Gut microbiota refers to a complex community of all types of microorganisms residing in the gastrointestinal tract in humans and other organisms in a mutualistic relationship. Although the majority of microorganisms belong to the category of bacteria, yet microorganisms of different classes, including fungi, viruses, protozoa, and archaebacteria are also found to be localized in the gut of humans [1,2]. Among bacteria, there are five main bacterial phyla present in the gut of human beings, and these include *Firmicutes, Bacteroidetes, Actinobacteria, Proteobacteria*, and *Verrucomicrobia.* Nevertheless, *Bacteroidetes and Firmicutes* constitute >90% of the total gut bacterial community [3,4]. From the physiological point of view, the relationship between humans and gut microbiota is mutualistic as humans provide shelter to microbiota and in return, microbiota helps in the synthesis of vital components (vitamin B and vitamin K), metabolism of certain compounds (bile, sterols, and xenobiotics) and fermentation of dietary products [5].

In recent years, the importance of gut microbiota has increased due to growing evidence of their involvement in human pathophysiology. Considering the critical role of these resident microorganisms in regulating the functions of different parts of the body and scientists have coined different terms, including the gut–brain axis [6], gut–kidney axis [7], gut–liver axis [8], and gut–heart axis [9]. It suggests that the effects of gut resident microorganisms are not localized to the gastrointestinal tract. Instead, the resident microbiota dynamically interacts with different organs in the body and regulates their functioning. The importance of gut microbiota in humans is further supported by the studies showing the linkage of gut microbiota with the pathogenesis of diseases including allergy, ulcerative colitis, diabetes mellitus, metabolic syndrome, Parkinson’s disease, and Alzheimer’s disease [10–12]. Apart from these, there have been studies documenting that gut microbiota may influence the cardiac functions, and their association with coronary artery disease (CAD) has been reported [13–16]. Coronary artery diseases constitute one of the significant causes of death and disability in developed countries. Indeed, CAD is responsible for one-third or more of all deaths in individuals over age 35 [14–16]. In CAD patients, a considerable change in the composition of gut microbiota has been identified. Gut microbe-mediated modulation of the cardiovascular diseases, including atherosclerosis and coronary artery disease, may be due to structural components of bacteria, including lipopolysaccharide or generation of diverse metabolites, including trimethylamine-N-oxide (TMAO), short-chain fatty acids, secondary bile acids, and uremic toxins like p-cresol sulfate and indoxyl sulfate. The data from the literature show that gut microbiota may be beneficial to the heart patients [15,17,18], or it may produce deleterious effects in CAD patients [19–21].

There have been some reviews describing the role of gut microbiota-derived metabolites in cardiovascular diseases. However, there are no reviews to explain the benefits as well as the harmful effects of gut microbiota in CAD. Therefore, this review aims to highlight that specific gut microbiota and their products may be deleterious, while other microbiota and their metabolites may produce beneficial effects in coronary artery disease. Accordingly, the present review discusses the change in the composition of gut microbiota in CAD patients, the useful role of microbiota (including probiotics) along with products such as short-chain fatty acids and secondary bile acid metabolites; harmful effects of gut microbiota along with their metabolites such as trimethylamine-N-oxide (TMAO), lipopolysaccharide (LPS), and uremic toxins. The review also discusses the conditions in which gut microbiota ore their metabolites may be useful or harmful.

## Gut microbiota composition and its possible use as a non-invasive parameter to diagnose CAD patients

There have been studies documenting that the alteration in the number/composition of gut-resident bacteria may be associated with the increased risk of development of CAD. In a retrospective study, the data from 1059 patients revealed that patients with small intestinal bacterial overgrowth had a higher frequency of CAD [22]. A comparative study conducted in CAD patients (*n* = 39) and healthy volunteers (*n* = 50) revealed a characteristic change in the gut microbiota. A significant increase of *Lactobacillales*, while a decrease of *Bacteroidetes* (*Bacteroides + Prevotella*) in the gut of CAD patients, was identified, suggesting the shift in microbiota may be a contributing factor in the development of CAD [23]. Another comparative study involving CAD patients (*n* = 29) and 35 healthy volunteers revealed a significant decrease in the proportion of *Bacteroidetes* and an increase in the proportion of *Firmicutes* in the gut of CAD patients [24]. Accordingly, it is proposed that the change in gut microbiota may be potentially employed as a diagnostic marker to identify CAD patients [25]. A recent study has also shown the changes in the composition of gut microbiota in CAD patients as compared with healthy persons. Overall, there was a significant reduction in the diversity and richness of gut microbiota in CAD patients. On a comparative scale, *Faecalibacterium* was the predominant species in healthy persons, whereas *Escherichia-Shigella* and *Enterococcus* species were more predominant in CAD patients. Other bacterial species that were relatively less represented in the gut of CAD patients included *Subdoligranulum, Roseburia*, and *Eubacterium rectale*. It further signifies that gut microbiota dysbiosis is an important risk factor for the development of CAD and identification of changes in its composition may be potentially employed for its diagnosis [26].

A clinical study compared the changes in the composition of gut microbiome along with functional alterations in atherosclerotic (*n* = 218) and healthy persons (*n* = 187). The results described the relative abundance of *Enterobacteriaceae*, including *Escherichia coli, Klebsiella* spp., *Enterobacter aerogenes* along with *Streptococcus* spp., *Lactobacillus salivarius, Solobacterium moorei*, and *Atopobium parvulum* in atherosclerotic patients. On the other hand, a relative reduction in *Bacteroides, Prevotella, Roseburia intestinalis*, and *Faecalibacterium cf. prausnitzii* was detected in atherosclerotic patients. Due to the change in the microbal composition, the functional changes were also observed in atherosclerotic patients. These functional changes included an increase in the transport of sugars and amino acids, along with a decrease in the biosynthesis of vitamins and tetrahydrofolate [27]. The beneficial role of *Bacteroides* species in the CAD was further confirmed in a clinical study showing a decrease in the abundance of *Bacteroides vulgatus* and *Bacteroides dorei* in the fecal matter of CAD patients [28]. More studies have shown the alteration in the gut microbiota in hypercholesterolemic patients (*n* = 15) in terms of an increase in the abundance of *Proteobacteria* and atherosclerosis-associated genus *Collinsella*. Atorvastatin is shown to increase the abundance of bacteria with anti-inflammatory properties such as *Faecalibacterium prausnitzii, Akkermansia muciniphila*, and *Oscillospira* in hypercholesterolemic patients (*n* = 27) [29].

An LC-MS-based metabolomics study identified N-acetyl-d-glucosamine 6-phosphate and l-carnitine (associated with an intestinal microbiota) as potential non-invasive biomarkers related to the progression of CAD [30]. Apart from the studies showing that gut microbiota is involved in the development of CAD, there may be a significant change in the gut microbiota following acute myocardial infarction. In the left anterior descending coronary artery ligation model, the abundance of *Synergistetes, Spirochaetes, Lachnospiraceae, Syntrophomonadaceae*, and *Tissierella Soehngenia* along with gut barrier impairment was identified. Therefore, an increase in microbiota following ischemia–reperfusion injury along with impairment in gut barrier may lead to an increase in the entry of toxic metabolites or bacterial products in the systemic circulation, which may contribute in increasing myocardial injury following myocardial ischemia [31].

## The beneficial role of microbiota/or their products in coronary artery disease patients

### Gut microbes/components reduce the risk of development of coronary artery disease

The role of intestinal microbiota in ischemia–reperfusion-induced myocardial injury was first reported by Russian scientists in 1986 [32]. Subsequently, in a prospective case–control study in middle-aged men from eastern Finland identified the correlation between enterolactone, a lignan produced by the intestinal microbiota from dietary precursors, and risk of development of CAD. Men with high enterolactone concentration (>30.1 nmol/l) have approximately 59% lower risk of acute coronary events. It suggests the importance of intestinal microbiota and plant-dominated fiber-rich food in lowering the risk of coronary heart diseases [17]. However, a case–cohort study in Finnish male smokers identified a weak association between serum enterolactone concentration and the risk of development of coronary heart disease [33]. It is possible that smoking attenuates the beneficial effects of enterolactone, and the latter may not able to confer cardioprotection in coronary artery disease patients (Table 1).

**Table 1:** Summarized effect of probiotics and antibiotics on the outcomes in preclinical and clinical studies related to coronary diseases

S. No	Interventions	Outcomes	References
**Probiotics**			
1.	Administration of *Lactobacillus rhamnosus* GG in high fat-fed and ApoE^−/−^ mice	Reduction in atherosclerotic plaque size and cholesterol levels	[39]
2.	Administration of *Lactobacillus rhamnosus* GG in high-fat subjected obese mice	Normalization of dyslipidemia, decrease in triglycerides, cholesterol and reduction subcutaneous adipose tissues	[40]
3.	Administration of *Lactobacillus rhamnosus* GR-1 in coronary artery occlusion model in rats	Attenuation of left ventricular hypertrophy, and improvement in systolic and diastolic left ventricular function	[14]
4.	Administration of *Lactobacillus plantarum* in diet-induced hypercholesterolemia model in mice	Decrease in cholesterol levels	[41]
5.	Administration of *Lactobacillus plantarum* 299v in CAD patients	Improvement in the vascular endothelial function	[15]
6.	Administration of *Bacteroides vulgatus* and *Bacteroides dorei* in atherosclerosis-prone mice	Attenuation of the development of atherosclerosis	[28]
**Antibiotics**			
1.	Maintenance of ApoE-KO mice in germ-free conditions (absence of microbiota)	More severe atherosclerosis	[77]
2.	Use of clarithromycin in CAD patients	Increased risk of mortality and morbidity	[79,80]

It has been shown that rat intestinal microbiota induces biotransformation of *Dioscorea nipponica* to derive diosgenin, which protects the myocardium against ischemic insult by increasing the levels of antioxidants and decreasing oxidative stress damage [34]. A study has ascribed the beneficial effects of mild exercise on the heart to changes in the composition of gut microbiota. In preclinical research, mice were subjected to mild exercise (on a treadmill) for 4 weeks before undergoing left coronary artery ligation, and changes in the gut microbiota were evaluated. It was reported that exercise training increased the relative abundance of *Butyricimonas* and *Akkermansia* in the gut and improved cardiac function in the ischemia–reperfusion model. Accordingly, it has been proposed that mild exercise may produce beneficial effects in coronary artery disease conditions by altering the composition of gut microbiota [18].

### Exogenous delivery of probiotics provide protection in coronary artery disease

Probiotics are the living microorganisms that produce various health benefits on consumption. There have been several clinical studies showing the beneficial effects of probiotics, including *Lactobacillus plantarum, Lactobacillus rhamnosus*, and *Lactobacillus curvatus* in terms of reducing the risk of development of coronary artery disease [35–38]. In high-fat fed apolipoprotein E knockout (ApoE^−/−^) mice, exogenous administration of *Lactobacillus rhamnosus* GG was shown to reduce atherosclerotic plaque, plasma adipocyte-fatty acid-binding protein (A-FABP) and cholesterol levels [39]. Another study also showed the efficacy of orally administrated *Lactobacillus rhamnosus* GG in normalizing dyslipidemia in high-fat fed obese mice, in terms of a decrease in the levels of triglycerides, cholesterol, along with the reduction in the weights of liver, mesenteric, and subcutaneous adipose tissues [40]. Moreover, administration of *Lactobacillus rhamnosus* GR-1 significantly attenuates the development of left ventricular hypertrophy and improves systolic and diastolic left ventricular function in coronary artery occlusion model in rats [14]. The hypocholesterolemic effects of *Lactobacillus plantarum* have also been reported in diet-induced hypercholesterolemia model in mice [41]. A very recent study has shown that daily administration of *Lactobacillus plantarum* 299v in patients of CAD for 6 weeks led to significant improvement in vascular endothelial function [15]. Based on the clinical finding showing the decrease in the abundance of *Bacteroides* in CAD patients, a preclinical study identified that oral gavage with live *B. vulgatus* and *B. dorei* attenuates the development of atherosclerosis in atherosclerosis-prone mice [28] (Table 2).

**Table 2:** Summarized description of potentially useful and harmful bacteria along with useful and harmful metabolites

S. No	Category	Bacteria	Metabolites	References
1.	Potentially Useful	*Butyricimonas, Akkermansia, Bacteroides, Prevotella*, and *Firmicutes*	• Enterolactone • Short chain fatty acids including acetate, butyrate and proprionate • Bile acids including deoxycholic acid, lithocholic acid, hyodeoxycholic acid and ursodeoxycholic acid	[17,18] [49,51,52] [58,59,60]
2.	Potentially Harmful	*Collinsella, Proteobacteria, Escherichia, Shigella*, and *Enterococcus*	• TMAO • Low molecular weight metabolites from aromatic amino acids • Lipopolysaccharide (LPS) and uremic toxins	[20,21] [19] [71,73]

The anti-atherosclerotic effects of these probiotics have been mainly attributed to the regulation of cholesterol levels [42], which may be due to an increase in cholesterol efflux and excretion from the enterocytes [43,44] along with the reduction in cholesterol absorption from the intestine [45]. The other possible contributing mechanisms may include a decrease in the release of lipopolysaccharide in the gut along with the suppression of pro-inflammatory immune response [28,36,46]; decrease in the levels of proinflammatory cytokines including IL-1β, IL-8, IL-12, and TNF-α [15,44]; reduction in the levels of TMAO [47]; increase in the bacterial diversity in the gut and alteration in the production of short-chain fatty acids [15,35,48]. The role of gut bacteria-derived-TMAO and short-chain fatty acids in CAD is discussed in the following section.

### Role of gut microbe-derived short-chain fatty acids in coronary artery disease

The microbial fermentation of dietary products such as carbohydrates and proteins leads to the generation of short-chain fatty acids including acetate, butyrate, and propionate [49]. These small fatty acid molecules may enter the portal circulation to activate G-protein coupled receptors, which may be responsible for the regulation of diverse functions, including innate immunity and host metabolism [50]. The beneficial effects of short-chain fatty acids in several disease conditions, including CAD, are well reported [51,52]. The cholesterol-reducing and improvement in endothelial functions in the presence of probiotics such as *Lactobacillus rhamnosus* and *Lactobacillus plantarum* are also attributed to an increase in the plasma levels of propionate [15,48]. In a very recent study, the anti-inflammatory properties of these molecules have been documented and a decrease in their levels has been associated with an increase in the development of atherosclerosis in mice [53] (Table 2).

### Role of secondary bile acid metabolites in coronary artery disease

Primary bile acids are synthesized by the hepatocytes and released from the gall bladder into the intestine, where these are acted upon by gut microbiota to produce secondary bile acids. These secondary bile acids may arise due to deconjugation of conjugated bile salts, dehydrogenation (oxidation of a hydroxy group to an oxo group), epimerization (conversion of α-hydroxyl group to β-hydroxyl group or vice versa), and removal of sulfate groups from the bile acids. Apart from these, 7α and 7β dehydoxylase may act to generate secondary bile acids including deoxycholic acid, lithocholic acid, hyodeoxycholic acid, and ursodeoxycholic acid [54,55] (Table 2). These secondary bile acids are potent anti-inflammatory molecules and these enter the portal circulation to trigger hormone-like signaling by acting on nuclear farnesoid X receptors (FXR) and pregnane X receptors (PXR) [51,56,57]. The activation of secondary bile acid receptors, i.e. FXR and TGR5, are associated with a decrease in the development of atherosclerosis in mice models [58,59]. Indeed in a clinical study, the serum lithocholic acid (the most powerful activator of TGR5) is considered as an independent predictor of coronary atheroma [60].

## Deleterious effects of gut microbiota in coronary artery disease

There have been studies documenting that the changes in the composition in gut microbiota, production of toxic metabolites, and related processes may produce harmful effects in CAD patients.

### Metabolites of gut microbiota produce toxicity to the heart

In a preclinical study, it has been reported that low molecular weight metabolites derived from intestinal microbiota are transported to the blood, and these may increase the severity of ischemia–reperfusion-induced myocardial injury. More precisely, it was reported that oral administration of non-absorbable, broad-spectrum antibiotics including vancomycin or a mixture of streptomycin, neomycin, polymyxin B, and bacitracin significantly decrease the severity of myocardial infarction. The decrease in myocardial infarction in response to antibiotic therapy was attributed to reduction in gut microbiota. Furthermore, it was shown that these animals had lower plasma levels of metabolites of aromatic amino acids, including phenylalanine, tryptophan, and tyrosine, which was possibly due to lower microbiota in the gut. This finding was supported by the results showing that exogenous administration of microbial metabolites of aromatic amino acids phenylalanine, tryptophan, and tyrosine attenuates the cardioprotective effects of non-absorbable, broad-spectrum antibiotics. Therefore, it may be proposed that gut microbiota may produce low molecular weight metabolites from aromatic amino acids, which may reach the systemic circulation to increase the susceptibility of ischemic myocardial damage [19].

### Role of trimethylamine-N-oxide (TMAO) in coronary artery disease

Trimethylamine-N-oxide (TMAO) is formed in the liver from trimethylamine, which is almost exclusively generated from dietary phosphatidylcholine by gut microbiota. Studies have shown the close association between TMAO and the development of CAD [20]. In Clinical Outcomes Utilizing Revascularization and Aggressive Drug Evaluation (COURAGE) trial-like patient cohort, a relationship between fasting plasma TMAO levels and mortality rate over 5 years in CAD patients (*n* = 2235) was investigated. About a 4-fold increase in mortality risk was identified in patients with higher plasma TMAO levels, suggesting that elevated plasma TMAO levels pose a significant risk among CAD patients [21]. Two independent cohorts, including the Cleveland cohort (*n*  =  530) and Swiss Cohort of ACS patients (*n*  =  1683) revealed the elevated plasma levels of TMAO, at the time of emergency presentation, as an independent risk factor for the development of major adverse cardiac events. They also reported that TMAO levels serve as predictors of long-term survival of CAD patients [61]. The meta-analysis from 17 clinical studies (*n* = 26,167), 4–5 years duration, revealed that the high TMAO plasma levels are associated with an increased incidence of all-cause mortality. There is around a 7.6% increase in mortality in response to every 10 μmol/l increase in TMAO levels suggesting the positive dose-dependent association between the TMAO levels and increased cardiovascular mortality [62]. Based on 3 years follow-up study in stable CAD patients (*n* = 3903), it was reported that subjects with the increased plasma level of choline and betaine (precursor of TMAO) are associated with 1.9- and 1.4-fold increased risk of major adverse cardiac events, respectively. Importantly, this increase in choline and betaine led to an increase in the risk of adverse cardiac events, only when there was an associated increase in plasma TMAO levels. It further signifies the importance of TMAO in producing deleterious effects on the heart [13]. In HIV-infected persons, an increase in plasma TMAO levels serves as a useful biomarker of cardiovascular risk after initiation of antiretroviral therapy [63].

A cross-sectional study in patients (*n* = 227) who underwent cardiovascular surgery showed a correlation between serum TMAO levels and the number of infarcted coronary arteries [64]. In another study in stable CAD patients (*n* = 353), a correlation was reported between cardiac troponin T and the fasting plasma TMAO levels suggesting that the fasting plasma TMAO levels are an independent predictor of a high atherosclerotic burden in CAD patients [16]. A very recent study on stable angina pectoris and acute coronary syndrome patients (*n* = 90) has revealed that the increased plasma TMAO levels make the atherosclerotic plaques more vulnerable to thin-cap fibroatheroma suggesting the increased risk of unfavorable cardiovascular events [65]. Accordingly, it may be proposed that gut microbe-generated metabolite TMAO, and may serve as a useful biomarker of CAD-related mortality. In contrast, the data obtained from retrospective healthy early middle-aged adults (*n* = 5115) in Coronary Artery Risk Development in Young Adults Study (CARDIA) trial revealed that the plasma levels of TMAO are not associated with early signs of development of atherosclerosis [66]. It might be possible to suggest that the elevated plasma levels of TMAO may not be deleterious in healthy humans, but these become critical in accelerating the disease progression in already existing heart disease (Table 2).

### Role of other gut microbiota-derived toxic mediators

Gut microbes may also contribute in inducing deleterious effects in CAD through lipopolysaccharide (LPS) and uremic toxins. LPS, also known as endotoxins, are the structural constituents of gram-negative bacteria, and gut microbes-derived LPS crosses the gut mucosa to enter into the systemic circulation. Studies have identified that LPS is one of the most potent activators of innate immune signaling [67]. It is also shown to trigger the development of allergy, autoimmune response [68], and chronic inflammation [69]. Due to its inflammatory [69] and vascular smooth muscle proliferating properties [70], its vital role in the pathogenesis of atherosclerosis has been defined [71]. Uremic toxins such as indoxyl sulfate, p-cresyl sulfate, and phenylacetylglutamine are derived from gut bacteria, which tend to accumulate in the body during a renal failure condition. Indeed, the rise in the plasma concentration of these uremic toxins is identified in persons with kidney failure and undergoing dialysis [72]. Furthermore, the increase in the plasma levels of these uremic toxins is reported to accelerate the development of atherosclerotic plaque [73] and increase the mortality rate [74].

## Role of antibiotics in coronary artery disease

Considering the deleterious effects of some of the gut microbiota, it has been hypothesized that the employment of antibiotics may produce beneficial effects in CAD. However, the results of clinical trials evaluating the efficacy of antibiotics in reducing mortality or cardiovascular events in CAD patients have been inconsistent [75,76]. Atherosclerosis prone ApoE-KO mice are shown to develop more severe atherosclerosis during germ-free conditions [77]. It is also reported that the employment of anti-microbial agents may produce harmful effects [78]. Although the treatment with antibiotics effectively modifies the gut microbiota composition, however, it does not reproduce the same experimental setting found in germ-free animals. There is an impaired metabolism, poor intestinal absorption, and immature immune response in germ-free mice [79]. Therefore, these points should be taken into account while comparing the results of germ-free mice and antibiotic-treated mice. Based on the negative results of double-blind trial (CLARICOR) evaluating the efficacy of clarithromycin in reducing the mortality in patients with stable CAD [80], FDA has issued a warning that use of the macrolide antibiotics may increase the risk of cardiovascular morbidity and mortality in patients with heart disease [81] (Table 1).

## Discussion

**Figure 1:**
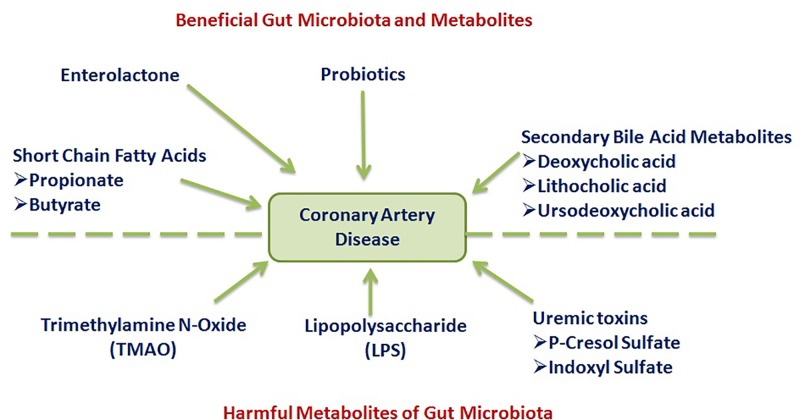
Gut microbiota may produce beneficial as well as deleterious effects in coronary artery disease patients, which is due to the production of useful or harmful metabolites

In recent years, there is an increase in the interest in gut resident microbiota due to the unfolding of their greater participation in human diseases [10–12]. In relation to CAD, studies have shown the beneficial as well as deleterious effects of gut microbiota [15–20]. The studies have shown that gut microorganisms may produce beneficial effects in the myocardial ischemic state either directly in the form of exogenous administration like *Lactobacillus* [14,15] or indirectly by acting on plant-dominated fiber-rich food to produce important components including enterolactone [33]. The metabolic products of gut bacteria including short-chain fatty acids such as propionate and butyrate [48,53] along with secondary bile acids such as deoxycholic acid, lithocholic acid, hyodeoxycholic acid, and ursodeoxycholic acid [54,60] produce beneficial effects in atherosclerosis and CAD (Figure 1). Furthermore, the cardioprotective effects secondary to mild exercise have been attributed to a significant change in microbiota composition, with a relative increase in colonies of *Butyricimonas* and *Akkermansia* in the gut [18].

However, there are abundant studies documenting the harmful and deleterious effects of gut microbiota on cardiovascular health, particularly in myocardial ischemia. Indeed, it has been reported that there is a significant alteration in gut microbiota in patients suffering from CAD [22,23]. A more significant finding has been the decrease of *Bacteroidetes* and increase of *Firmicutes, Escherichia–Shigella* and *Enterococcus* in the gut of CAD patients suggesting that the shift in microbiota may be one of contributing factor in the development of coronary heart disease [23,24,26]. It is possible to suggest that due to alterations in the profile of gut microbiota, there are changes in the metabolism in the gut and subsequently, altered metabolic products may gain entry in the blood circulation to produce deleterious effects. It has been reported that there are changes in the metabolism of aromatic amino acids and small molecular weight metabolic products reach in the systemic circulation to produce harmful effects to the heart [19]. The most widely studies toxic metabolite arising due to abnormal metabolic activity of gut microbiota is TMAO, whose plasma levels have been directly correlated to the risk of major cardiovascular events in CAD patients [21,62,65]. Consequently, many studies have proposed fasting plasma TMAO levels as a biomarker to predict the risk of development of adverse cardiovascular events [16,63]. Other harmful components/metabolites derived from gut microbes include LPS [70] and uremic toxins, including indoxyl sulfate, p-cresyl sulfate (Figure 1), and phenylacetylglutamine [73]. The plasma levels of these uremic toxins tend to rise, particularly in patients suffering from kidney disease or undergoing dialysis [72]. It is an important point to note that most of the findings related to the effects of microbiota on CAD have come from the clinical studies, and there are not enough preclinical studies to describe the actions of microbially derived molecules at the molecular level.

Based on these studies, it may be proposed that there are benefits as well as deleterious effects of gut resident microbiota in CAD. As long as the gut microbiota works “normally” and maintains a balance (symbiosis), the host physiology is maintained, and protective effects of these bacteria are obtained. On the other hand, the disturbance in their growth pattern in the form of selective outgrowth of certain bacteria and restrictive growth of others tends to produce dysbiosis, which is deleterious for the host. A significant shift in the composition in the gut microbiota is a deleterious and selective decrease in beneficial microorganisms (*Bacteroides, Prevotella*, and *Firmicutes*) and an increase in the colonies of harmful microbiota (*Collinsella, Proteobacteria*, and *Enterobacteriaceae*) contributes in the development of CAD. The exogenous delivery of probiotics may be used to compensate for the selective decrease in the number of beneficial bacteria occurring in CAD. However, selective restriction on the growth of harmful bacteria with the help of antibiotics seems difficult as antibiotics also tend to kill the useful bacterial colonies and there is more harm to the individual. Nevertheless, selective inhibition of LPS or uremic toxins metabolites may potentially yield beneficial effects to overcome the deleterious effects of harmful gut microbiota.

## Conclusion

Gut microbiota may have beneficial or harmful effects in cardiovascular disease patients. The change in the composition of gut microbiota in CAD patients may predispose the patients to more detrimental effects, and patients become more prone to major adverse cardiovascular events. A selective decrease in beneficial microorganisms including *Bacteroides, Prevotella*, and *Firmicutes* and an increase in the colonies of harmful microbiota including *Collinsella, Proteobacteria*, and *Enterobacteriaceae* may contribute to the development of CAD. The bacteria-derived metabolites, including enterolactone; short-chain fatty acids including acetate, butyrate and propionate; bile acids including deoxycholic acid, lithocholic acid, hyodeoxycholic acid, and ursodeoxycholic acid are useful and produce beneficial effects. On the other hand, TMAO, low molecular weight metabolites from aromatic amino acids, lipopolysaccharide, and uremic toxins are deleterious metabolites and produce harmful effects. Exogenous delivery of probiotics or pharmacological inhibition of harmful products of gut microbiota may potentially overcome the deleterious effects in a coronary artery disease condition.

## Abbreviations

CAD: coronary artery diseases
FXR: farnesoid X receptors
LPS: lipopolysaccharide
PXR: pregnane X receptors
TMAO: trimethylamine-N-oxide
